# Bipolar I disorder: a qualitative study of the viewpoints of the family members of patients on the nature of the disorder and pharmacological treatment non-adherence

**DOI:** 10.1186/s12888-020-03008-x

**Published:** 2021-02-08

**Authors:** Nasim Mousavi, Marzieh Norozpour, Zahra Taherifar, Morteza Naserbakht, Amir Shabani

**Affiliations:** 1 Department of Clinical Psychology, University of Social Welfare and Rehabilitation Sciences, Tehran, Islamic Republic of Iran; 2 Department of Psychology, Faculty of Psychology and Educational Sciences, University of Tehran, Tehran, Islamic Republic of Iran; 3 Faculty of Behavioral Sciences and Mental Health, Tehran Psychiatry Institute, Iran University of Medical Sciences, Tehran, Islamic Republic of Iran

**Keywords:** Bipolar I disorder, Treatment non-adherence, Family psychological education, Qualitative study

## Abstract

**Background:**

Bipolar disorder is a common psychiatric disorder with a massive psychological and social burden. Research indicates that treatment adherence is not good in these patients. The families’ knowledge about the disorder is fundamental for managing their patients’ disorder. The purpose of the present study was to investigate the knowledge of the family members of a sample of Iranian patients with bipolar I disorder (BD-I) and to explore the potential reasons for treatment non-adherence.

**Methods:**

This study was conducted by qualitative content analysis. In-depth interviews were held and open-coding inductive analysis was performed. A thematic content analysis was used for the qualitative data analysis.

**Results:**

The viewpoints of the family members of the patients were categorized in five themes, including knowledge about the disorder, information about the medications, information about the treatment and the respective role of the family, reasons for pharmacological treatment non-adherence, and strategies applied by families to enhance treatment adherence in the patients. The research findings showed that the family members did not have enough information about the nature of BD-I, which they attributed to their lack of training on the disorder. The families did not know what caused the recurrence of the disorder and did not have sufficient knowledge about its prescribed medications and treatments. Also, most families did not know about the etiology of the disorder.

**Conclusion:**

The lack of knowledge among the family members of patients with BD-I can have a significant impact on relapse and treatment non-adherence. These issues need to be further emphasized in the training of patients’ families. The present findings can be used to re-design the guidelines and protocols in a way to improve treatment adherence and avoid the relapse of BD-I symptoms.

## Background

Bipolar I disorder (BD-I) is a chronic and recurrent psychiatric disorder in which a person has a manic episode for 1 week, which may present before or after hypomanic or major depressive episodes [1].

BD-I is accompanied by chronic stress, disability, increased risk of sudden mood swings, higher rates of comorbid disorders and moral, financial, and legal problems. The disorder is ranked the sixth debilitating disease according to the World Health Organization (WHO). BD-I is considered the most expensive mental disorder in terms of the health and behavioral care required by the patients and the burden on governmental institutions and insurance companies [2–4]. According to a report by the Central Bank of the Islamic Republic of Iran, the average annual income of an Iranian household in 2012 was 209,050,000 Rials. The direct annual cost of one BD-I patient consists of 10% of this average family income [5].

BD-I affects the patient’s life and has long-term consequences that are visible in the patient’s social performance and quality of life [6, 7]. Severe impairment in job performance is observed in about 30% of the patients with BD-I. In such cases, functional improvement falls substantially behind symptom improvement [1].

Pharmacological treatment is the first-line treatment for BD-I. Evidence shows that about 40% of patients with BD-I do not have good medication adherence, which translates into a higher probability of symptom relapse, hospitalization, and increased suicide risk [8]. In a study in Tehran, Iran, poor treatment adherence was noticed in about 30% of BD-I patients [9]. Another study from Iran [10] also reported the prevalence of poor compliance in BD-I patients after the first episode of mania as 38.1% during a 17-month follow-up period. Therefore, it is of great importance to better understand and investigate the underlying reasons for treatment non-adherence in BD-I patients.

Given the changes implemented in health care systems over the last two decades and the resultant focus on community-based services, the role of families in caring for BD-I patients has become more prominent [6]. The insufficient knowledge of families about the disorder, its symptoms, and medications has made the management of BD-I more difficult and eventually imposes additional costs on them [6]. The higher is the cost imposed on the family, the more likely is it for the family members to show adverse reactions to the BD-I patients, which itself leads to a higher chance of disorder relapse [3].

In Iran, the general public is acquainted with various types of psychiatric illnesses through mass media and public educational websites such as the website of the Iranian Psychiatric Association (https://iranmentalhealth.com) and other Persian public written sources. Patients with BD-I and their families become familiar with the treatment process after consulting a general practitioner, a psychiatrist, or a psychologist, and, if necessary, the patients are admitted to the hospital through a psychiatrist. In addition to medical treatment, they receive the necessary training and information about their treatment process in the hospital. Furthermore, an association called ABR (Association of Mental Health Promotion), with an active website (http://abrcharity.ir), independently monitors patients, including those with bipolar disorder, after discharge.

Many studies have examined the views and roles of patients with BD-I and their caregivers and also the importance of family awareness and its impact on medication adherence. Tacchi & Scott [11] and Veligan et al. [12] suggest that the family members’ beliefs about the nature of BD-I and the information they have about the disorder affect the patient’s medication adherence. The review of literature showed no precise studies conducted to explore the knowledge, information, and opinions of family members of BD-I patients about the disorder and the causes of their medication non-adherence.

In a previous study in Iran [13], the authors held qualitative interviews with the family members of patients with BD-I and reported that treatment non-adherence is a major problem in these patients. They also reported that the patients and their families did not have sufficient knowledge about the nature of this disorder. Considering these findings about the insufficient knowledge of the family members of BD-I patients and the high rate of treatment non-adherence, it is necessary to conduct more studies to investigate the possible causes of treatment non-adherence and families’ knowledge and beliefs about this disorder in Iran. This study was thus carried out to explore the viewpoints of the family members of BD-I patients about the nature of this disorder and the potential causes of treatment non-adherence. The results can be used for revising the psychoeducation guidelines for BD-I patients, as clinical guidelines mandate the inclusion of psychoeducation in the treatment plan adopted for these patients. The results can also be used to design a protocol to address the disorder relapse, which can have substantial consequences in terms of reducing healthcare costs.

## Methods

The findings of this study are reported according to the Consolidated Criteria for Reporting Qualitative Research (COREQ) checklist [14].

### Study samples’ characteristics

The participants were the family members of patients diagnosed with BD-I. The patients had been admitted to Iran Psychiatry Hospital in Tehran, Iran, and were receiving pharmacological treatments.

This study used purposive sampling to select the participants. From November 2017 to April 2018, 12 patients were interviewed by two psychiatrists based on the DSM-5 criteria [1] and received the diagnosis of BD-I. Then these diagnoses were confirmed by A.SH. and their families were invited to participate in the study.

None of the family members refused to participate in the study and they all completed the entire course of the study. The mean age of the participants was 50.83 years. There were three male (25%) and nine female (75%) participants (Table 1). Table 2 shows further details on patients’ characteristics.

**Table 1:** Participants’ demographic characteristics

Participants (*n* = 12)	
Age, years: mean (SD) range	50.83 (9.73) 36–67
Male: n (%)	3 (25)
Marital status, n (%)	
Single	2 (16.7)
Married	9 (75)
Widowed	1 (8.3)
Education, n (%)	
Middle school or lower	5 (41.67)
High school	2 (16.7)
Bachelor’s	3 (25)
Master’s or higher	2 (16.8)
Relationship with the patient, n (%)	
Patient’s mother	7 (58.3)
Patient’s son	1 (8.3)
Patient’s wife	1 (8.3)
Patient’s husband	1 (8.3)
Patient’s sister	1 (8.3)
Patient’s brother	1 (8.3)

**Table 2:** patients’ demographic and clinical characteristics

Patient code	Interviewed patient’s relative code	Patient’s relationship with the interviewee	Gender	Age	Marital status	Occupation	Duration of the disorder	People living with the patient
1	1	Daughter	Female	20–30	Single	Unemployed	8 years	Parents
2	2	Son	Male	30–40	Single	Unemployed	10 years	Parents
3	3	Son	Male	30–40	Single	Unemployed	14 years	Mother
4	4	Father	Male	60–70	Divorced	Unemployed	12 years	Nobody
5	5	Husband	Male	30–40	Married	Supermarket salesperson	3 years	Parents and husband
5	10	Son	Male	30–40	Married		3 years	Parents and wife
6	6	Wife	Female	30–40	Married	Housewife	10 years	Husband and two children
7	7	Son	Male	20–30	Single	Unemployed	10 years	Mother
8	8	Son	Male	20–30	Single	Unemployed	4 years	Parents and brother
9	9	Son	Male		Single	Unemployed		
10	11	Brother	Male	30–40	Single	Unemployed	16 years	Mother and sister
11	12	Brother	Male	30–40	Single	Real Estate Advisor	8 years	Parents

### Data collection

After diagnosing patients with BD-I, and obtaining the written consent of the family members of patients to participate in this study, data were collected by in-depth interviews from family members of patients, conducted at the hospital’s conference hall. No one else was present at the time of the interviews except for the interviewer and the participant. Each interview lasted approximately 20 min and was digitally recorded for subsequent analyses. Two female PhD candidates (N. M. and M. N.) in clinical psychology at the University of Social Welfare and Rehabilitation Sciences, Tehran, Iran, who had already received training on the implementation of qualitative studies, held the interviews. They did not know any of the participants. The interviewers introduced themselves to the participants before the beginning of each interview. The interview questions were provided by the authors. The interviews were held only once and were not repeated. Data saturation was reached with 12 participants, and no further participants were interviewed after reaching this number. Data saturation occurs when no new information is obtained by conducting further interviews [15].

### Data analysis

Thematic analysis was used for the qualitative data analysis [16, 17]. To this end, the six steps proposed by Clark and Brown [17] were used.

The raw data derived from the interviews were used for the analysis. The content of the interviews were transcribed verbatim immediately after each interview. Field notes were made during the interviews and were reviewed in this stage. Three authors (M. N., N. M., and Z. T.) read the interviews several times for immersing in the data and getting familiar with it. Line-by-line coding was then applied to generate the initial codes. These steps were performed manually by the three authors without using any computer programs. One author encoded each interview and the interview was then read by another author and encoded again. The individually-extracted codes were then integrated and modified, if necessary.

In the next step, by linking the codes together, their common patterns and concepts were extracted and potential themes and subthemes were identified, keeping the research questions in mind. The data related to the themes were then collected and examined to verify the accuracy of the themes and subthemes, which resulted in five final themes.

Several statements were selected from the interviews as examples and are reported in the results section. To preserve participants’ anonymity, their names and ages are not mentioned in the results; instead, they are represented by random numbers.

## Results

Taking into account comprehensiveness, homogeneity, and overlap, the components of the family members’ viewpoints on the nature of the disorder and the reasons for pharmacological treatment non-adherence were categorized into five themes, including knowledge about the disorder, information about the medications, information about the treatment and the respective role of the family, reasons for pharmacological treatment non-adherence, and strategies applied by families to enhance treatment adherence in the patients.

Each of the themes contained several subthemes, which were themselves made up of some open codes. These subthemes contained recurrent codes and concepts that shared a common meaning.

Table 3 presents the themes, subthemes and examples of some of the codes.

**Table 3:** Categories of perspectives of families with type one bipolar disorder concerning the nature of the disease and non-adherence to treatment

Order	Themes	Subthemes	Code examples^a^
1	knowledge about the disorder	General knowledge	Not knowing the name of the disorder Knowledge of mania phase Knowledge of depression phase Not mentioning depression phase Lack of knowledge about the outcome of the disorder Misunderstanding recovery and medication effects
		Knowledge about signs and symptoms of mania phase	Aggression Labile mood Irritability Agitation Running away from home Battering Tendency to leave home Insomnia Grandeur delusion Having two personality states and Assuming another person identity
		Knowledge about signs and symptoms of depression phase	Anorexia Isolation Suicidal ideation Insomnia Loss of libido Ideation of dying
		Knowledge about etiology of disorder	Genetic abnormality Family treatment during childhood Family not being guilty Lack of proper training of parents before parenthood Unusual conditions of parents during sexual intercourse Brain disorder Mother’s lack of understanding the patient during puberty Divorcing the spouse Genetic abnormality in addition to precipitating factors Insufficient etiological knowledge about disorder
		Knowledge about the relapse reasons of disorder)	Discontinuation of medications Unemployment and society’s lack of support Financial problems Death of the father
2	Information about the medications	Knowledge about effects of the administered medications on patient	Reduction in aggression No reduction in aggression Reduction in the patient’s omnipotence Patient’s mood stability Reduction in running away from home Loss of libido Reduced lying Cessation of self-talking No effect on job functioning
		Knowledge about the side effects of prescribed medications	Medication addiction Obesity and increased appetite Increased sleep Reduced activity and energy Gastrointestinal complications Headache Loss of memory Hump Loss of vivacity Loss of fluency Sluggish speaking Difficulty waking up
3	Information about the treatment	Knowledge about the treatment	The need to take medications These patients’ incomplete and lasting recovery Treatment: the patient being employed Treatment: exercising The patient’s need to be monitored by the doctor The medication advantages outweigh its disadvantages Treatment: The patient participation in educational classes and development of insight about the disease The family’s negative perspective on medication Futility of psychologist and counseling Treatment: childbearing (wrong) Treatment: things progressing according to the patient’s wish
4	Information about the role of the family in the treatment		Calming the patient and sympathizing and empathizing with him Not stimulating the patient Bearing with the patient Not leaving the patient alone Encouraging the patient to exercise Encouraging the patient to go out Normalization of disorder Generating hope Taking the patient to the doctor Making the house quiet Breaking up the patient’s drug-use implements Informing significant others life of patient’s symptoms Tricking the patient into hospitalization Comforting the patient and creating a sense guilt in them
5	Reasons for pharmacologic treatment non-adherence		Relatives’ comments Comments of the powerful and knowledgeable people The patient’s worsening symptoms The patient missing their medications The patient’s stubbornness and anger toward the family The family’s non-familiarity with the disorder and the treatment process The patient’s negative ideations about medications Medication side-effects affecting the patient’s appearance Medication side-effects affecting the patient’s functioning Permanent change of the patient’s doctor by the family TV celebrity talks about futility of medications Not visiting the doctor during new year holidays The patient not being annoyed by the symptoms during mania phase Using alcohol Lack of insight about their own disorder Absence of a family member to help the patient with taking medications Family not agreeing to look after the patient The patient not waking to take their medications Relative’s disagreement with the patient and their stubbornness

^a^To keep the Table brief, only some repetitive codes in the subthemes are mentioned in the column called *code examples*

### Theme one: knowledge about the disorder

Most interviewees did not have sufficient or accurate knowledge about the nature of BD-I, the signs and symptoms of depression and mania cycles, and the outcome of the disorder. They mentioned the lack of training or inadequate training (especially by healthcare providers) as the main cause of insufficient knowledge about BD-I. Additionally, most families did not have a good understanding of the etiology of BD-I.

Some of them considered BD-I as a genetic abnormality, while others considered factors such as adolescent maltreatment, parents’ unusual conditions during sexual intercourse, and the lack of proper training before parenthood as potential causes of BD-I.

Participant No. 5 (a patient’s wife): *“I was told that he has a nervous problem.”*

Participant No. 3 (a patient‘s mother): *"I have a theory about having babies. I think that not everyone should have children. The husband and wife should be screened and monitored for two years to see if they understand the matter clearly. Do you see these anomalies now? ... These shameful movies they watch … The person is not feeling well when raising their kid … From an Islamic point of view, from a human’s point of view, both the husband and wife need to be monitored. Their food and other things should also be monitored to see if they can have a healthy baby.”*

Participant No. 7 (a patient’s mother): *"Because this boy is always impressed by me, sometimes I tell myself, maybe I didn’t fully understand him during his puberty. Sometimes I blame myself, as he has said this many times. I always blame myself … .*

*Sometimes he says, ‘You did this to me, that’s why I’m sick now and take drugs’. For example, when hitting puberty, in the first or second year of high school, he used to get up late and so he got to school very late. Then the school’s principal complained to me, ‘Why is he late again?’ And he says, ‘Why did you wake me up early in the morning? You did this to me.”*

Participant No. 10 (a patient’s mother), referring to her son's divorce: *"That's why he's so broken.”*

Participant No. 11 (a patient’s sister): *"Bipolar disorder has a genetic background. I think there would be no one out there who suffered from the disorder unless they got the genes. It is a genetic disorder, but it emerges when a patient experiences a series of shocking events. Well, some have higher potentials, such as those who get very angry. I mean, the anger itself is not part of the disorder, but in angry people, shocking events affect the patient more rapidly.”*

### Theme two: information about the medications

Many family members had a misconception about the treatment of the disorder and the effects of psychotropic medications on the patients. In other words, they were unable to accurately identify the therapeutic effects of the administered medications and the time it took for the patients to show signs of improvement. Also, some participants were unaware of the side-effects of the prescribed medications. Some mentioned side-effects like memory loss and drug addiction; however, almost all the participants believed that pharmacological treatment is necessary for the patients despite the side-effects.

Participant No. 1 (a patient’s mother): *"The problem of her running away from home with her boyfriend was a big burden for us, but as the prescribed meds began to show their effectiveness, this problem was gradually solved and we finally managed to put up with her aggressiveness and other problems. That is, we were saying to ourselves, ‘This is a period of angriness; we had better not said this, not done that’... We thought the medication was working. But now they’ve told me, ‘No, your patient has not recovered at all, has not been cured.”*

Participant No. 1 (a patient's mother): *"Her first psychiatrist, who has been visiting her for eight years, was frequently asking if she studies, watches TV or goes to work at all. ‘Whenever she goes back to these routines, then she has recovered,’ the therapist would say. Recently, she’s always been saying, ‘I would love to go to work’ and so on. Once, her employer told her to do some cleaning, and she had responded, ‘I’m not your servant.’ She suddenly broke it off and said, ‘I won’t go to work anymore.’ She didn’t sleep at all, saying, ‘I work so much, but I don’t feel exhausted at all.’ We were also excited and thought ‘Yeah, so this doctor's meds have been good; she’s getting back to normal, she’s working,’ She was frequently organizing her closet, like an obsession.”*

Participant No. 3 (a patient’s mother): *“I can’t remember the side-effects but I’ve heard about them in classes. My daughter is taking lithium now but she gets these chills. Her stomach is not well. Its side-effects are such that they affect her memory. However, when we compare the pros and cons, we have to take it. "*

### Theme three: information about the treatment

The regular intake of medications, stress control, work, exercise, regular visits to a psychiatrist or psychologist, and the need to provide insight into the patient’s illness through education were noted by the families in this part. Some participants believed that psychotherapy sessions cannot help treat this disorder while some had completely false or superstitious beliefs about treatment of the disorder.

Participant No. 4 (a patient's son): *"Our patient doesn’t accept justifications. When you bring them to classes and convince them that ‘You are sick, and you have to take this medication because of this and that, and we have evidence that you have this disorder,’ and then we show it to them, prove it like in the movies, say that this disorder is serious because of so and so reasons, I think, it would be much easier.”*

Participant No. 1 (a patient’s mother): *"They sent us to get counseling. Of course, my daughter did not cooperate and didn’t come with. So, I got an appointment under my name to get information and find out how to deal with this disorder. Then the psychologist said, 'No, your daughter is diagnosed with bipolar disorder; this is an acute illness. Counseling does not work for her. She should take medications –a lot of them. And since the doc said those words, we withdrew from counseling altogether.”*

Participant No. 5 (a patient’s wife): *"My mother-in-law says, ‘If God gives him a baby, he’ll be fine.’ Because his ex-wife also failed to bear a child for him.”*

### Theme four: information about the role of the family in the treatment

Most families defined their role as helping the patient recover and adhere to their treatment, reminding them to take the medications, encouraging them to go to the doctor, not leaving them alone, and doing whatever they wanted to do so that things went as the patient wished. The patients also appeared to feel guilty when their families tried to comfort them, and this pattern was observed in several of the participants in this study.

Participant No. 6 (a patient’s husband): *"We should put up with her, love her, not argue about what she says, listen to her, get her to do exercise to keep busy. I'm here now and I brought her with me too instead of leaving her alone to think about stuff.”*

Participant No. 2 (a patient’s mother): *"You should be good to them, listen to them, make home a peaceful environment, and not argue.”*

Participant No. 8 (a patient’s wife): *“I don't know. If he just thinks that everything is okay, all will be okay; but such feelings don’t last forever.”*

Participant No. 2 (a patient’s mother): *"I tell him to take his meds on time … Say, ‘Let's go to the park to take a look around ... Don't stay at home too much. God is merciful; it won’t be that bad’ … I talk to him, I comfort him sometimes, tell him that I’m ill too because I feel your pain.’ I really do. I’ve been crying alone at home many times. God, what will happen at the end?"(She cries).*

### Theme five: reasons for pharmacological treatment non-adherence

As for this theme, the participants noted issues that were mostly about the comments made by other people, including relatives or care-providers, such as doctors or specialists in other disciplines. An interesting observation was made by a participant who mentioned a celebrity talking on TV about the inefficiency of medications; following these comments, the patient had stopped taking his medications. Another issue was that the families’ constant changing of the patient’s physician contributed to their medication non-adherence. Another reason noted for non-adherence was that the patients did not suffer from mania symptoms and found that it was not so crucial for them to take the medications. Additionally, some patients reported the physical discomfort and weakness (e.g., impotence) experienced as side-effects of the prescribed medications a reason for their medication non-adherence.

Participant No. 2 (a patient’s mother): *"She didn't take the meds for seven to eight months. Her friend had told her ‘Your eyes look different. When you take the medicine, your eyes turn into a strange shape. Get rid of them.’ After seven months, her disease relapsed.”*

Participant No. 6 (a patient’s husband): *"If we go to a party somewhere and someone asks her, ‘Oh, you take drugs?’ … But that person is not aware of the matter, cause she might look all well, and that person doesn’t know what’s actually happening in my wife’s mind, who then has to admit that she is alright."*

Participant No. 7 (a patient’s mother): *"At one point at work, some colleagues told him, ‘You will become addicted to the medicines, you will get sick.’ Then, he put the medicines aside and became pessimistic about his work. ‘This job has made me sick,’ so he said and left his job all of a sudden. He had a great job, not a difficult one. He could manage it by himself very easily.”*

Participant No. 3 (a patient’s mother): *“My son had gone to a doctor to remove the corn on his feet. The doctor had checked his medicine prescriptions and asked, ‘What are these you’re taking? You won’t be able to conceive a baby in the future. It’ll affect you poorly’ and so on. My son keeps repeating what the doctor told him.”*

Participant No. 1 (a patient’s mother): *"That emergency nurse who came to our house told us to change her doctor. Since then, she has kept repeating this sentence. She threw out all her medicines.”*

Participant No. 3 (a patient’s mother): *“Since the beginning of the new year, he’s began to no longer take his medications. In Khandevaneh,*^1^*Mr. Mehran Ghafourian (a famous Iranian actor) said, ‘I was in a bad mood ... I had depression. I put the medications aside and started exercising.’ My son stopped taking his medicines on hearing those words. I asked him many times to go see a doctor but he said no. He continued to not take his medicines and then his disorder worsened. He was frequently beating us up until we took him to the hospital with the help of the police.”*

Participant No. 3 (a patient’s mother): *“There was a child psychiatrist on a TV talk. We took our son to her office. We used to visit a counselor as well. The psychiatrist prescribed him some medications. We didn’t know what the medications were. He was taking his medicines. In the middle of therapy, we stopped it. Then, my son-in-law, who is a doctor, said ‘Dr. A -his professor- is a very good doctor.’ My son used to go to Dr. A. earlier when he was a college student. He was taking medicines and he believed in him so much. Then again, my eldest daughter, who is a physician, said ‘Dr. B. is a very helpful therapist. All the doctors, engineers, and educated people go to visit him.’ Then he went there ... And two years ago, I took him to Dr. S. too, to help him get rid of his substance abuse."* (This participant named seven different doctors).

Participant No. 4 (a patient’s son) discussed the reasons for the patient’s refusal to take the medications and said: *"Well, he doesn't actually believe in the disorder being a real one (in the manic episode). Maybe now he takes the pill in front of you, but you know that it is not something that bothers him. You take pills more easily if you have actual pain, but when you don’t, you ask yourself ‘Why do I have to take all these pills?”*

Participant No. 11 (a patient’s sister): *“We can note the poor behaviors of those around him. He considers any weaknesses he experiences (e.g., sexual problems) a side-effect of the medicines he’s taking. And he’s linking everything to the medicines and thinking they’re going to make him different from the others.”*

## Discussion

The findings of this study regarding the viewpoints of the family members of patients with BD-I were categorized into five themes. Although qualitative studies do not allow for the identification of the extent and relative importance of every condition, recurrent themes and concepts stated by the participants at different individual and social levels were extracted.

Research suggests that there is a relationship between families’ knowledge and beliefs about the disorder and the patients’ medication adherence [12]. The attitudes and knowledge of the family members have a significant influence on the patient’s own beliefs and attitudes and affect the patient’s decision about treatment compliance [18]. In agreement with previous studies [19, 20], the family caretakers in this study were shown to lack sufficient information and knowledge about the nature of BD-I. In addition, many participants had inaccurate or false information and insisted on these false beliefs. A review study on treatment acceptance found that brief interventions focused on relapse prevention and psychoeducation-based interventions have the greatest impact on relapse prevention [21]. Maintaining the patients’ circadian rhythms (especially sleep rhythm), controlling activity levels, verifying and controlling initial symptoms of mania and depressive episodes, and not using narcotics or stimulants have been recommended in approved psychotherapy protocols for bipolar patients [22]. Nonetheless, the participants in this study did not discuss any of these important factors. The lack of knowledge about these important issues among families can have a significant impact on relapse and treatment non-adherence in the patients. These points need to be further emphasized in training patients’ families.

In a qualitative study on bipolar patients and their families, Peters, Pontin, Lobban, and Morriss [23] found that the viewpoints of patients and their families play an important role in managing the disorder; however, the families usually get despondent about participating in this process, and their perception was that some mental health workers believe that family involvement makes their work more complicated. Meanwhile, the present study showed that, in Iran, families do not have enough information about their role in preventing disorder relapse and attribute their patient’s relapse only to factors such as medication withdrawal, unemployment, lack of community support, and financial problems. Most of them believed that if everything goes as the patient wishes, the disorder will not relapse.

Furthermore, the participants did not have adequate information about the non-pharmacological treatment options available for this disorder and the role that psychologists can play in helping the patients enhance their medication adherence and prevent the symptoms of relapse. A variety of behavioral, cognitive, and emotion-focused interventions are used in the management of bipolar disorders [22]. Nevertheless, the participants did not have sufficient knowledge about these treatments. The observation that many psychologists in Iran appear unwilling to participate in the treatment of bipolar disorder patients seems to play a role in this lack of knowledge. According to Farhoudian et al. [24], only about 1.5% of all the studies on psychiatric disorders conducted in Iran between 1973 and 2003 involved bipolar and cyclothymic patients. In a qualitative study on bipolar-II patients and their families, Fisher et al. [25] found that the number of resources available to patients for deciding about their treatment has increased and their priorities have been given increasing attention; yet, the patients’ and their families’ preferences are not fully considered.

Similar to the studies carried out by Jönsson, Wijk, Skärsäter & Danielson [26] and Shamsaei, Mohamad Khan Kermanshahi, and Vanaki [27], in the present study, the patients and their families were struggling with the acceptance, understanding, and management of the disorder. According to the participants, the families’ lack of insight into the patients’ disorder contributed significantly to their medication non-adherence. This finding is in line with Scott and Pope’s [28] research, but Delmas, Proudfoot, Parker, and Manicavasagar [29] stated that the rejection of treatment is a complex issue that depends on various factors.

Some of the results of this study are consistent with the findings reported by Clatworthy, Bowskill, Rank, Parham, and Horne [8], who noted that deliberate treatment non-adherence is associated with factors such as patients’ concerns about the prescribed medications and their side-effects in the case of continuous consumption. Proudfoot et al. [30] stated that the side-effects of medications, coping with unpleasant symptoms, the extent of awareness about the nature of the disorder, and the reactions to it as well as the stigma associated with the disorder affect the patient’s life path. Besides, these symptoms have a permanent impact on the disorder relapse [31]. The findings showed that the interaction of the disorder, patient, medications, psychiatric attitude, and cultural attitude with non-compliance is very complex [32].

In addition to the themes mentioned, there were some interesting results concerning the response process in all the interviews. For example, the majority of the participants only reported symptoms of the manic episode, while two major studies [33, 34] have shown that people with bipolar I and II (especially type II) disorders spend most of their symptomatic days with depression. Patients suffer greatly during the depressive episodes but have elevated or irritable moods during the manic episode; in contrast, families find the mania symptoms more annoying and disruptive to themselves. This duality can negatively impact reaching a common understanding with the patient about visiting the doctor and taking medications. Moreover, the fact that some families do not have enough information about the depressive episode can eventuate in neglecting the patient’s need to take medications during this phase, which can then adversely affect medication adherence. These results are somewhat contradictory to the results of a previous study [29], which reported that both patients and their family members report symptoms of mania and hypomania to their physicians less often, as some of them enjoy the manic symptoms. Family members feel relieved when they see that their patient is happy and shows mania symptoms. A major cause of this discrepancy in findings may be the differences in the study populations. While Delmas et al. [29] studied patients with bipolar I and II, the present study examined only patients with BD-I. The discrepancy may also partially originate from cultural differences. It seems that when there is a pattern of greater attention to objective and apparent symptoms, very important mental symptoms such as suicidal thoughts, whether during the mania or depressive episode, are neglected by families.

This study showed that families with a higher educational and socioeconomic status tend to seek psychiatric care from different psychiatrists. Frequently changing the treating psychiatrist can cause treatment non-adherence in the patients. Furthermore, as the family members of such patients falsely think that they have greater medical information, they are more likely to encourage the patient to stop taking their prescribed medications.

A major limitation of this study was that most participants were the mothers of the patients, as it was hard to find other family members of the patients to participate in the study. For example, only one child of a patient and one sister were among the participants. Also, all the participants were from Tehran and were selected from one hospital; therefore, the generalization of the results to other cities in Iran should be pursued with caution.

## Conclusion

The authors suggest using the findings of this qualitative study regarding the knowledge of the family members of patients with bipolar I disorder (BD-I) as well as the dominating cultural beliefs to design further quantitative studies. The quantitative assessment of individual, familial, and social reasons for treatment non-adherence is also a recommendation for future research. Conducting similar studies on the family members of patients with other types of bipolar disorder with an attention to the different processes and outcomes involved is also recommended. Since there are different ethnicities and subcultures in Iran, the results obtained by examining the residents of the country’s capital city cannot be generalized to the population of other cities and towns, and it is necessary to repeat the study in other populations in order to get familiar with other viewpoints in Iran.

Overall, the results of this study contribute to the emerging qualitative research on bipolar disorder and provide the readers with an insight into the viewpoints of the family members of patients with BD-I. Some inaccurate information might have been observed in participants’ statements due to some deeply-rooted cultural attitudes and beliefs and their correction may require extensive interventions.

The results of this study can be used to compile educational content for patients with bipolar disorder and their families as well as for psychologists, psychiatrists, psychiatry assistants, and hospital health workers.

## Data Availability

The datasets used and/or analyzed during the current study are available from the corresponding author on reasonable request.

## Footnotes

1 A popular comedy show on Iranian television
